# Human Plasma Metabolomics Identify 9-cis-retinoic Acid and Dehydrophytosphingosine Levels as Novel biomarkers for Early Ventricular Fibrillation after ST-elevated Myocardial Infarction

**DOI:** 10.1080/21655979.2022.2027067

**Published:** 2022-01-30

**Authors:** Jieying Luo, Junaid Ahmed Shaikh, Lei Huang, Lei Zhang, Shahid Iqbal, Yu Wang, Bojiang Liu, Quan Zhou, Aisha Ajmal, Maryam Rizvi, Maryam Ajmal, Yingwu Liu

**Affiliations:** a Department of Heart Center, The Third Central Hospital of Tianjin, Tianjin, China; b Tianjin Key Laboratory of Extracorporeal Life Support for Critical Diseases, Tianjin, China; c Artificial Cell Engineering Technology Research Center, Tianjin, China; d Faculty of Life Science and Medicine, Tianjin Institute of Hepatobiliary Disease, Tianjin, China; e GKT School of Medical Education, Faculty of Life Science and Medicine, King’s College London, London SE1 IUL, UK; f Department of Clinical Laboratory, Tianjin Third Central Hospital, Tianjin, China; g St George’s Hospital Medical School, St. George’s, University of London, Cranmer Terrace, London, SW17 0RE UK

**Keywords:** Acute myocardial infarction, acute coronary syndrome, ventricular fibrillation, metabonomics, sudden death

## Abstract

The relevant metabolite biomarkers for risk prediction of early onset of ventricular fibrillation (VF) after ST-segment elevation myocardial infarction (STEMI) remain unstudied. Here, we aimed to identify these imetabolites and the important metabolic pathways involved, and explore whether these metabolites could be used as predictors for the phenotype. Plasma samples were obtained retrospectively from a propensity-score matched cohort including 42 STEMI patients (21 consecutive VF and 21 non-VF). Ultra-performance liquid chromatography and mass spectrometry in combination with a comprehensive analysis of metabolomic data using Metaboanalyst 5.0 version were performed. As a result, the retinal metabolism pathway proved to be the most discriminative for the VF phenotype. Furthermore, 9-cis-Retinoic acid (9cRA) and dehydrophytosphingosine proved to be the most discriminative biomarkers. Biomarker analysis through receiver operating characteristic (ROC) curve showed the 2-metabolite biomarker panel yielding an area under the curve (AUC) of 0.836. The model based on Monte Carlo cross-validation found that 9cRA had the greatest probability of appearing in the predictive panel of biomarkers in the model. Validation of model efficiency based on an ROC curve showed that the combination model constructed by 9cRA and dehydrophytosphingosine had a good predictive value for early-onset VF after STEMI, and the AUC was 0.884 (95% CI 0.714–1). Conclusively, the retinol metabolism pathway was the most powerful pathway for differentiating the post-STEMI VF phenotype. 9cRA was the most important predictive biomarker of VF, and a plasma biomarker panel made up of two metabolites, may help to build a potent predictive model for VF.

## Introduction

With the increasing popularity of primary percutaneous coronary intervention technology and the shortening of reperfusion time, an increasing number of ST-elevated myocardial infarction (STEMI) patients can receive timely coronary revascularization and benefit from an improved long-term prognosis. However, the in-hospital mortality rate of these patients has not decreased significantly in the past decade [1]. One of the contributing factors is the sudden cardiac death caused by ventricular fibrillation (VF) in the early stage after acute myocardial infarction. This is especially in the case of STEMI as it usually precedes the availability of medical aid and accounts for most of the death [2]. Even for in-hospital cardiac arrest with bystanders, the subsequent rescue treatment will inevitably consume significant medical resources. Therefore, the risk stratification of early VF in patients with acute coronary syndrome has important social and health economic significance.

The search for predisposing factors has attracted a wide range of studies recently. Previous studies suggest a genetic predisposition may significantly contribute to the vulnerability of the ischemic myocardium to VF [3]. Some observations demonstrated that larger infarct size or lower levels of omega-3 polyunsaturated fatty acids are correlated with a higher incidence of primary VF in acute myocardial infarction patients [4,5]. There are, however, several conflicting results regarding the correlation between these susceptibility factors and the phenotype in clinical practice and animal models [5–7]. This suggests that the relationship between these predisposing factors and arrhythmogenesis may be relatively weak, hence alluding to the presence of additional contributing factors.

Metabolomics is a discipline developed after genomics and proteomics: an important component of systems biology. By detecting the variations of terminal metabolites within a biological system, the different responses of organisms to a variety of internal and external environmental disturbances can be determined [8]. Some technical means, such as ultra-performance liquid chromatography and mass spectrometry (UPLC/MS) help diagnose or monitor the progression of a disease, or a reaction to medication, all through samples of body fluid and feces [9–11]. In our previous studies, by analyzing the plasma metabolites of patients with myocardial infarction, we successfully constructed a model that distinguished the pathological condition of STEMI as well as age, and identified sphingolipid metabolism as the topmost changed pathway in young STEMI patients [12]. However, as far as we know, potential biomarkers based on metabolomics to predict the early occurrence of VF after myocardial infarction have not been reported.

**Figure 1.**
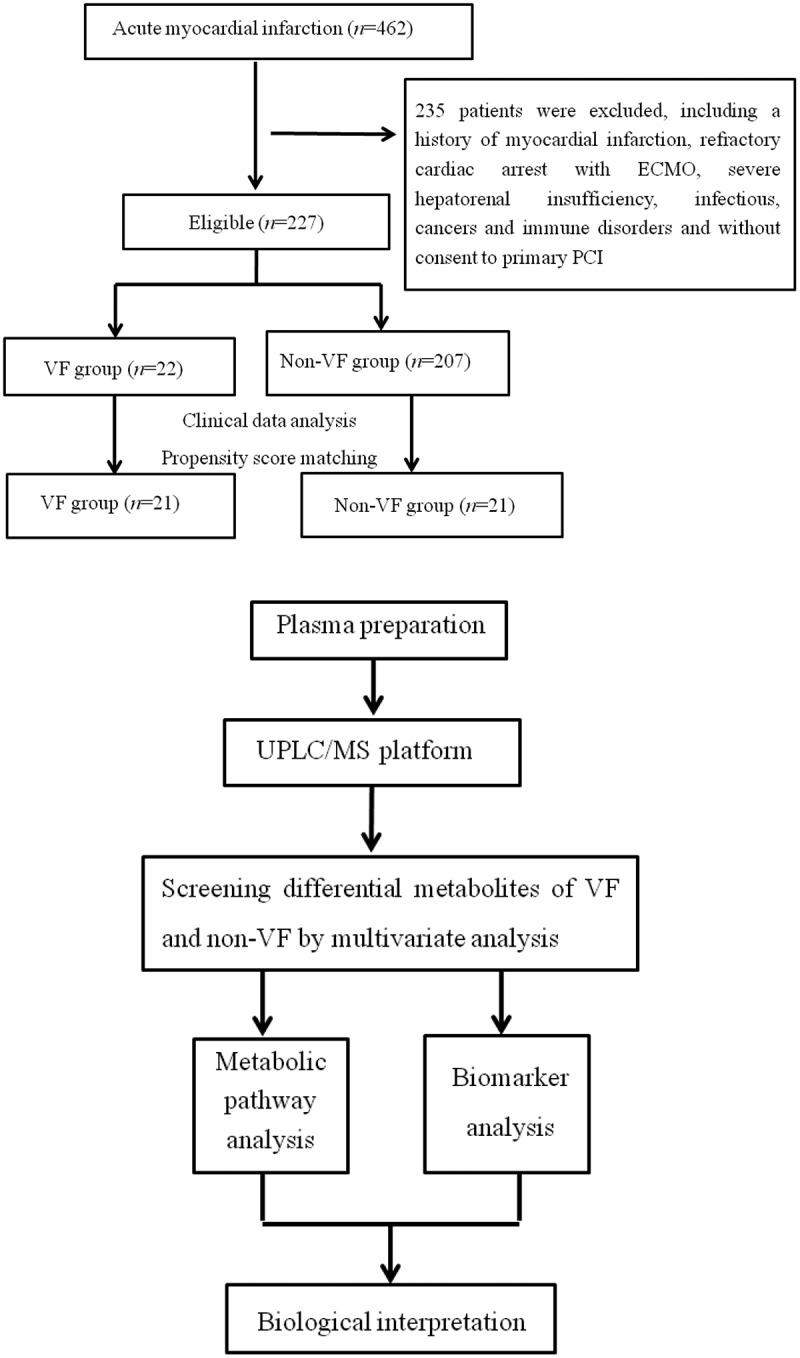
Schematic flowchart of Patient selection and metabolic profiling strategy used in this study. UPLC/MS, ultra-performance liquid chromatography and mass spectrometry; ECMO extracorporeal membrane oxygenation, VF ventricular fibrillation; PCI, percutaneous coronary intervention.

Until recently, using UPLC/MS again, we were surprised to find that the proportion of early episodes of VF in the left main coronary artery disease (LMCAD) group was significantly lower than that in the non-LMCAD group after adjustment for clinical variables by propensity score matching (4/22 vs. 17/22, *P* < 0.001). This indicated that the etiology of early VF onset after STEMI might be another underlying unknown risk factor independent of the LMCAD phenotype. Thus, changes in the profile of metabolites in the blood may help to identify the potential causes. Against this hypothesis, we aimed to identify some characteristic changes in plasma metabolites and capture valuable biomarkers with good discrimination for the early VF episode after STEMI. A flowchart of the overall experimental design is illustrated in Figure 1.

## Patients and Methods

### Patients

**Inclusion criteria**: 1) Age 18–75, 2) The diagnosis of STEMI was based on the third universal definition of STEMI symptoms [13], and all subjects enrolled received primary percutaneous coronary intervention and standard medical therapy thereafter. 3) As for the VF group, all events of VF have been confirmed by the electrocardiogram monitor and occurred prior to revascularization. **Exclusion criteria**: 1) refused percutaneous coronary intervention, 2) history of myocardial infarction, ischemic or non-ischemic cardiomyopathy, malignant arrhythmia, or dysfunction of multiple organs.

On the basis of this pilot data set, the MetaboAnalyst tool was used to estimate sample size. The specific calculation method refers to our previously published literature [14]. According to the calculations, the study’s robustness (about 0.95) would be achieved with approximately 20 samples in each group. The 227 STEMI patients enrolled in this study were from a total of 462 STEMI inpatients and age-sex-matched healthy volunteers from 1 January 2016 to 13 May 2017, who were used to conduct our previous metabolomic analysis of LMCAD [14]. Early onset of VF occurred in 22 of the patients, who were defined as the VF group, and the remaining 205 cases were assigned to the non-VF group. To ensure homogeneity of all clinical characteristics apart from the phenotype of VF, a propensity score matched non-VF group was selected for our disease control group.

### Ethics approval

This study was conducted according to the Declaration of Helsinki and was approved by ethics committees from Tianjin Third Central Hospital.

### Methods for Calculating Propensity Scores

Propensity score matching (PSM) was used to reduce selection bias and to balance baseline characteristics that could influence the episodes of VF. The PSM between the VF and non-VF STEMI groups was created using propensity score estimates obtained via a logistic regression model for the presence of VF as a function of the following parameters: comorbidities, symptoms-to-balloon interval, grading of cardiac function, culprit vessel, Gensini score, mean arterial pressure, white blood cell, serum levels of creatinine, potassium, magnesium, B-type brain natriuretic peptide, D-dimer at admission and peak of myocardial enzymes. We used nearest-neighbor matching to do a 1-to-1 PSM analysis, using a caliper value of 0.2 standard deviation of pooled propensity scores. Patients who were not satisfactorily matched within the accepted rank range were excluded.

### UPLC/MS sample preparation

As the purpose of our study is to find out the differential metabolites of early VF after STEMI, the time point of blood collection was set to immediately after the diagnosis of STEMI. 3 mL of venous blood was collected from patients’ peripheral veins and stored in a tube containing anticoagulant of ethylenediamine tetraacetic acid. Blood samples were centrifuged at 1600 × g for 10 minutes to extract plasma, after which they were kept at −80°C until analysis. Before analysis, a 100 µL plasma sample from each patient was thawed at room temperature and then mixed with 400 µL of methanol and then subjected to 30 seconds of strong vibration, followed by a 5-minute incubation at 4°C for protein precipitation. After that, the mixture was centrifuged for 30 minutes at 4°C at 15,000 × g. The supernatant was evaporated and redissolved with a 5% acetonitrile aqueous solution, then filtered through a 0.22 µm membrane, and analyzed.

**Sample analysis** An Accela system, which was equipped with a binary solvent delivery manager and a sample manager, was utilized to perform chromatography. As described in our previous literature [12,14], a Thermo Hypersil GOLD C18 reverse-phase column was employed as the analytical column. 0.1% formic acid aqueous solution (phase A) and 95% acetonitrile with 0.1% formic acid (phase B) were used in the UPLC mobile phase. The chromatographic extraction period for each sample was 15 minutes. The injection volume of each sample was 10 μL with a flow rate of 200 μL/min maintained throughout. Temperatures for the column oven and sample manager were set to 20°C and 4°C, respectively. The chromatographic elution gradient was started at 5% phase B and maintained for 3 minutes. Phase B was linearly intensified to 50% over the course of 10 minutes, then a quick climb to 95% was achieved in 3 minutes. Phase B was promptly lowered to 5% after maintaining that high volume of organic phase gradient for 4 minutes, and this elution gradient was employed to equipoise the analytical column for the last 4 minutes.

A cation-mode LTQ Orbitrap XL hybrid mass spectrometer was used for mass spectrometry (MS), with a capillary voltage of 30 V, an ion source voltage of 4.5 kV, a cone voltage of 150 V, a sheath gas flow of 30 arb, a desolvation temperature of 275°C, and an assistant gas flow of 5 arb (99.999% nitrogen). The data were taken in centroid mode over a 15-minute period, with the range of mass-to-charge ratio (m/z) of 50–1000. The resolution of the MS was set to a million (full width half maximum [FWHM]). Thermo Fisher Scientific provided all the calibration standards (caffeine, Ultramark 1621, and MRFA). Collision-induced dissociation with a normalized collision energy of 35% was used in tandem mass spectrometric (MS/MS) analysis. 99.9999% helium was present in the collision gas.

**Quality control** Equal aliquots from each sample were blended together to make a quality control solution. Before we ran any test samples, ten consecutive quality control samples were injected, and the remaining quality control samples were added after every ten samples were assessed [15]. Cross-contamination was avoided by adding a blank between neighboring samples, which was created randomly using Microsoft Excel’s random number generator.

### Data processing and analysis

The data obtained from the UPLC-LTQ Orbitrap XL platform analysis were directly imported into MZmine 2.0 software (http://mzmine.sourceforge.net/). The open-source software for LC-MS data processing was used to determine peak detection, alignment, and normalization (taking the total ionic strength of each sample as the standardization factor). Ionic mass (m/z), retention time, and peak intensity were rearranged from LC/MS experimental data into a matrix [16]. A chromatographic peak intensity signal-to-noise ratio >30, a retention time tolerance of 0.1 min, and a m/z tolerance of 0.01 were the filter criteria. The 80% rule was used to remove the variable with too many missing values, and MZmine was used to assign values to the variables with fewer missing values. The data table obtained from the analysis was a two-dimensional peak table (I × J). Each row (I) represents a sample, each column (J) represents a parameter, i.e., M/Z, and the numerical value represents the ion peak intensity (peak integral area). After that, the information was entered into the SIMCA-P 12.0 software package (Umetrics, Umea, Sweden). After mean centering and Pareto scaling between the groups under each condition, disease-distinguishing models of principal component analysis (PCA) and orthogonal partial least squares discrimination analysis (OPLS-DA) were created and cross-validated. The variables with the variable influence on projection score > 1.0 were deemed relevant for group discrimination. The variable influence on projection value and S-plot were used to obtain the significant variables for subsequent metabolism pathway analysis. The permutation test was used to confirm no overfitting in the study when the intercept of R2 in the Y-axis was less than 0.4, and that of Q2 in the Y-axis was less than 0.05.

### The identification of the characteristic metabolites

On the basis of the preliminary screening variables, we reset ten parameters of the apparatus. The secondary mass spectrometry (MS2) of the metabolites was determined using a MS2 scan of the control solution. The chosen ions were decided first by comparing the exact m/z value and retention time extracted from the MS2 ion chromatogram with the authentic reference standards, e.g. the human metabolome database (http://www.hmdb.ca) [16]. For the rest, the retrieved substance with a m/z deviation less than 0.02 was supposed for further confirmation if the ionization modes were identical to those in the human metabolome database. The characteristics of ions according to MS2 spectra were compared with those of theoretical fragments used in the preliminary results to ensure that MS2 m/z deviated remained less than 0.2, there was matching in the top three peaks, and the m/z deviated remained less than 0.2. Multivariate analysis was applied to the resulting two-dimensional matrix, which included sample names, m/z pairs, retention time and ion peak intensity [16].

### Statistics analysis

SPSS Statistics 23.0 software (IBM, Chicago, IL, USA) was utilized for the analysis of the clinical data. Normally distributed quantitative data, expressed as a mean ± standard deviation, were compared via the *t* test or *t′* test appropriately between two groups. Median and interquartile ranges were used to express non-normally distributed quantitative data, and the Mann-Whitney *U* tests were used to compare them. The frequency and composition of qualitative data were reported and the difference between two groups was calculated using Fisher’s exact test. All p-values were two-sided and were considered to be statistically significant when *p* < 0.05. MetaboAnalyst 5.0 version (Wishart Research Group, Canada), free online software, was used to undertake metabolite pathway and biomarker studies [16]. To ensure the reliability of differential metabolite screening, several multivariate algorithms were applied, including univariate and multivariate ROC curve analysis, linear Support Vector Machine (SVM) classification algorithm and Monte Carlo cross-validation.

## RESULTS

VF in the early phase of STEMI is a devastating complication that cannot be simply attributed to the size of myocardial infarction or the location of coronary lesions (e.g., LMCAD). We conducted this single-center retrospective case-control study to determine if there are other risk factors for morbidity. Here, plasma samples were collected immediately after admission from two cohorts of patients with STEMI (early VF group and non-VF group) generated by propensity score matching, and their non-targeted metabolite profiles were detected by UPLC/MS and were analyzed comparatively.

### Baseline characteristics of the groups before and after PSM

Two hundred and twenty-seven eligible patients were enrolled in the study, including 22 early VF episodes (VF group) and 205 non-VF patients (non-VF group). A one-to-one PSM produced 21 pairs. Twenty-one healthy control volunteers matched by gender and age in the same enrollment period were included. Table 1 shows a comparison of baseline characteristics between VF and non-VF groups before and after PSM. Before PSM, the VF group, compared with the non-VF group, was demonstrated to have worse cardiac function on admission and a higher proportion of left main coronary artery and left anterior descending branches in culprit vessels. Additionally, cardiopulmonary resuscitation in the catheter lab and intra-aortic balloon insertion in the VF group were also more common compared to the non-VF group, which led to a worse survival discharge rate.

**Table 1.** Baseline characteristics of the patients in the unmatched and propensity-matched groups

	Unmatched Groups			Matched Groups		
Variable	VF (*n* = 22)	Non-VF (*n* = 205)	*p*	VF (*n* = 21)	Non-VF (*n* = 21)	*p*
Age	61(52.5,73.3)	62(55,55,71)	0.907	62.0(54.0,73.5)	62.0(56.5,79.0)	0.562
Male, *n*(%)	18(81.8)	153(74.6)	0.458	17(81)	16(76.2)	1.000
BMI (kg/m^2^)	24.2(21.5,26.0)	24.8(22.5,27.2)	0.154	23.5 ± 3.1	24.7 ± 2.9	0.211
Killip			<0.001			0.116
I	8(36.4)	186(90.7)		8(38.1)	15(71.4)	
II	5(22.7)	16(7.8)		5(23.8)	4(19.0)	
III	5(22.7)	2(1.0)		5(23.8)	1(4.8)	
IV	4(8.2)	1(0.5)		3(14.3)	1(4.8)	
Coronary heart disease, *n*(%)	3(13.6)	29(14.1)	1.000	3(14.3)	4(360)	1.000
Hypertension, *n*(%)	8(36.4)	111(54.1)	0.112	8(38.1)	14(66.7)	0.121
Diabetes, *n*(%)	6(27.3)	49(23.9)	0.726	5(23.8)	8(38.1)	0.505
Cerebral infarction, *n*(%)	3(13.6)	19(9.3)	0.510	3(14.3)	3(14.3)	1.000
Syndrome to Balloon (h)	4.5(3.7,5.4)	4.8(3.6,7.9)	0.421	4.5(3.7,5.5)	6.0(3.7,9.9)	0.138
MBP (mmHg)*	88.2 ± 13.3	100.2 ± 16.6	0.001	88.2 ± 13.3	97.2 ± 16.2	0.051
LMCAD, *n*(%)	3(13.6)	19(9.3)	0.510	3(14.3)	3(14.3)	1.000
Ge*n*si*n*i score	53.5(37.8,82.5)	56(37,82)	0.925	61.8 ± 27.0	52.2 ± 27.3	0.260
Culprit vessel, *n*(%)			0.020			0.198
LAD	16(72.7)	100(48.8)		15(71.4)	10(47.6)	
Lcx	0(0)	23(11.2)		0(0)	1(4.8)	
RCA	5(22.7)	81(39.5)		5(23.8)	10(47.6)	
LM	1(4.5)	1(0.5)		1(4.8)	0(0)	
Number of vessels involved			0.692			0.920
1	6(27.3)	41(20.0)		6(28.6)	7(33.3)	
2	7(31.8)	65(31.7)		6(28.6)	5(23.8)	
3	9(10.9)	99(48.3)		9(42.9)	9(42.9)	
CPR in Cath lab *n*(%)	7(31.8)	5(2.4)	<0.001	6(28.6)	1(4.8)	0.093
IABP *n*(%)	5(22.7)	6(2.9)	<0.001	4(19.0)	1(4.8)	0.343

BMI body mass index, MAP mean arterial pressure, LAD left ascending descending artery, Lcx Left circumflex artery, RCA right coronary artery, CPR cardiopulmonary resuscitation, IABP intraaortic balloon pulsation

In terms of laboratory examination data (Table 2), the VF group had a higher white blood cell count and higher levels of D-dimer, serum creatinine and glucose (The *p-*values of the latter two tend to be significant). It is noteworthy that no significant difference between the two groups was observed in the peak value of myocardial enzyme and serum potassium level on admission. Nevertheless, all the baseline characteristics were equalized after PSM.

**Table 2.** Laboratory examination of the patients in the unmatched and propensity-matched groups

	Unmatched Groups			Matched Groups		
Variable	VF (*n* = 22)	Non-VF (*n* = 205)	*p*	VF (*n* = 21)	Non-VF (*n* = 21)	*p*
WBC(×10^9^/L)*	13.0(8.3,17.4)	9.5(7.9,11.5)	0.003	12.9(8.0,17.5)	10.5(8.2,12.3)	0.131
hemoglobin (g/L)*	149.3 ± 15.9	145.6 ± 16.7	0.326	147.9 ± 15.0	146.4 ± 16.5	0.763
Albumin (g/L)	38.2 ± 4.1	39.1 ± 3.2	0.216	38.4 ± 4.1	39.6 ± 2.5	0.297
Glucose (mmol/L)*	9.9(7.3,14.2)	8.1(6.78,10.2)	0.074	9.0(7.3,14.0)	7.8(6.9,9.9)	0.239
sCr (umol/L)*	77.2(64.4,94.4)	69.0(59.8,79.9)	0.059	79.5 ± 20.8	78.5 ± 30.8	0.901
Uric acid (umol/L)	375.5(262.8,469.8)	301.5(244.8,349.3)	0.004	384.3 ± 140.3	313.8 ± 83.9	0.057
Potassium (mmol/L)*	3.89 ± 0.55	3.80 ± 0.46	0.219	3.88 ± 0.56	3.90 ± 0.55	0.917
Magnesium (mmol/L)*	0.91 ± 0.12	0.90 ± 0.13	0.476	0.91 ± 0.12	0.90 ± 0.13	0.634
CKMB (U/L)	147(81,422)	176(98,288)	0.742	137.0(80.0,438.5)	199(94,288)	0.850
TG (mmol/L)	1.12(0.95,1.91)	1.36(0.98,1.91)	0.375	1.23(0.93,2.00)	1.47(0.99,2.83)	0.285
CHO (mmol/L)	4.82(3.99,5.35)	4.69(4.10,5.42)	0.679	4.81 ± 1.27	4.68 ± 0.93	0.720
BNP (pg/ml)	52.0(21.5,248.0)	36.2(13.0,116.0)	0.278	51.9(21.1,186.0)	11.5(5.0,261.0)	0.208
HbA1C (%)	6.1(5.6,8.5)	5.9(5.6,6.6)	0.274	6.0(5.6,8.0)	5.9(5.9,6.5)	0.714
hsCRP (mg/L)	5.77(1.53,12.60)	4.15(1.46,9.42)	0.525	5.8(1.5,12.6)	3.1(1.7,9.2)	0.546
LDL-C (mg/L)	3.09 ± 0.87	3.02 ± 0.82	0.737	3.08 ± 0.85	3.11 ± 0.77	0.924
D-dimer (mg/L)*	0.11(0.1,0.22)	0.1(0.1,0.1)	0.003	0.12(0.10,0.24)	0.10(0.10,0.18)	0.243

All the items marked with asterisk were the test results immediately after admission. a key factor of PMS analysis between the groups WBC white blood cell, sCr serum creatinine, CKMB Creatine kinase-MB isoenzyme, TG triglyceride, CHO Cholesterol,bnp B-type brain natriuretic peptide, HbA1C glycosylated hemoglobin A1C, hsCRP High sensitivity C-reactive protein, LDL-C Low density lipoprotein cholesterol

### Univariate and multivariate analyses of characteristic metabolites to distinguish VF and non-VF groups

**Figure 2.**
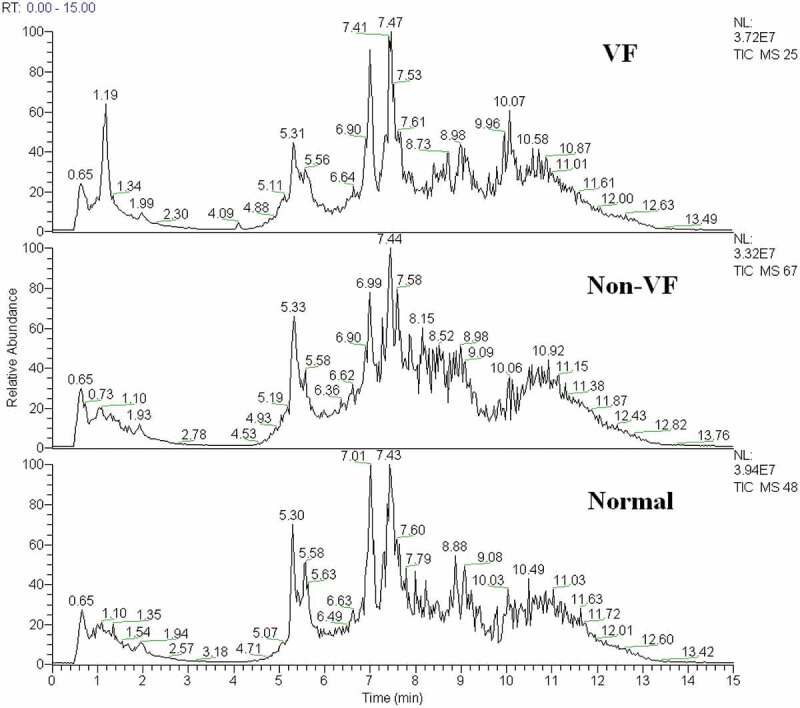
The total ion chromatogram of the metabolic profiles in different groups was obtained from SIMCA-P 12.0 (one sample chosen randomly). VF: group with ventricular fibrillation; non-VF: group without VF.

A typical total ion current chromatogram from a randomly selected sample in UPLC/MS is represented in Figure 2, where a significant difference can be detected between the VF and non-VF chromatograms. Multivariate analysis of the quality control samples revealed that the peak area deviation was within two standard deviations, confirming that the analytical results were reliable.

**Figure 3.**
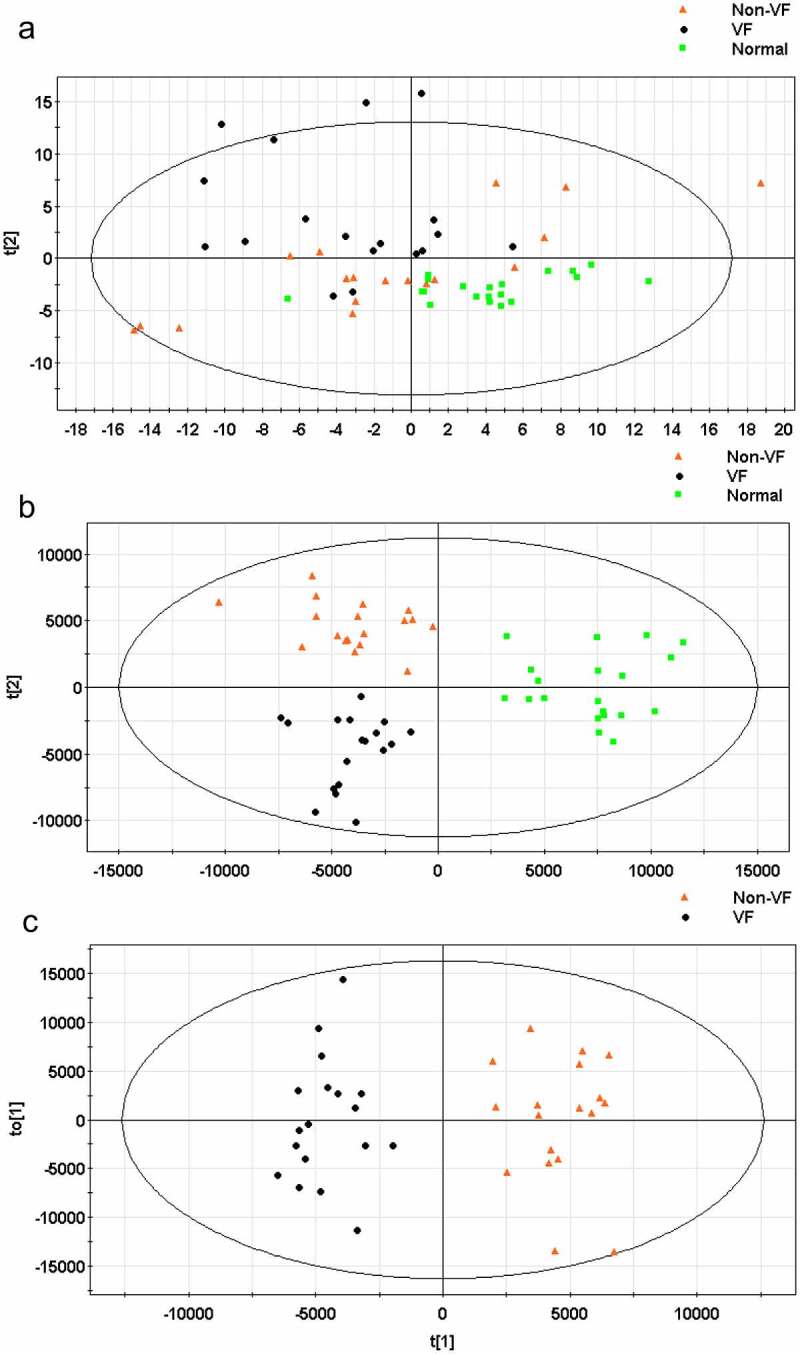
(a) The score plot for the first two principal components was shown. (b) OPLS-DA score plot with two predictive principal components and six orthogonal principal components was built (R2X = 71.1%, R2Y = 79.9%, Q2 = 46%) for all participants (VF, non-VF and healthy control (normal). (c) OPLS-DA score plot of the VF (VF) and non-VF (non-VF) group. One predictive principal component and four orthogonal principal components (R2X = 51%, R2Y = 92.4%, Q2 = 60.3%). Notes: All figures were developed from SIMCA-P 12.0 and every point represents a sample. R2 scores suggest performance of a model, and Q2 scores is an assessment of reproducibility, based on cross-validation.

After preprocessing and standardizing the data, a principal component analysis (PCA) model was developed with nine principal components (R2X = 53.2%, Q2 = 1.01%) for all participants (VF, non-VF, and healthy control). The score plot for the first two principal components is shown in Figure 3a, from which we could not conclude that there is a tendency of distinguishing clustering among groups.

Furthermore, an orthogonal partial least squares discriminant analysis (OPLS-DA) model was applied to magnify the subtle differences. The model produced a better discrimination into each cluster with two predictive principal components and six orthogonal principal components (R2X = 71.1%, R2Y = 79.9%, Q2 = 46%) for all participants (VF, non-VF, and healthy control) (Figure 3b). Here, R2X and R2Y represent the interpretation rates of the model to the X and Y matrices, respectively. Q2 represents the prediction ability of the model. The closer their values are to 1, the better the fitting degree of the model. When only post-MI VF and non-VF groups were compared, in the direction of the first predicted principal component (X-axis), the clustering differentiation between the two groups had a more significant trend, with one predictive principal component and four orthogonal principal components (R2X = 51%, R2Y = 92.4%, and Q2 = 60.3%) (Figure 3c). This suggests that the OPLS-DA model can be applied to distinguish the specific population.

### Comparison of Characteristic metabolites among groups

**Figure 4.**
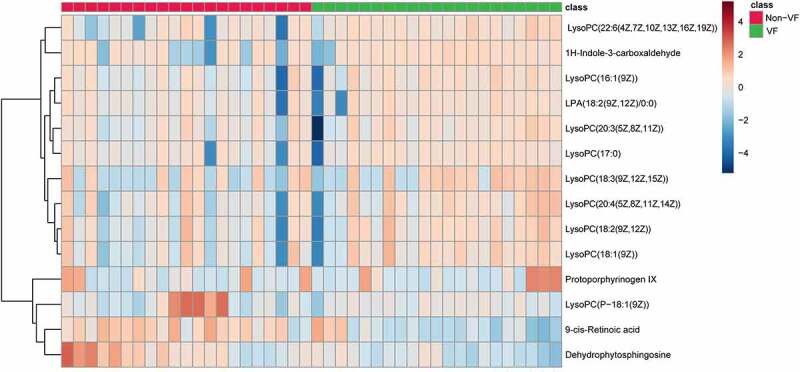
Heat map of differential metabolites in VF and non-VF groups demonstrate hierarchical clustering of altered metabolites in clustering analysis in different groups.

A total of 14 metabolites play an important role in distinguishing VF and non-VF groups. They were determined by a two-component OPLS-DA model of the UPLC/MS data sets (Table 3). A heatmap plot was constructed based on the differential metabolites correspondingly, from which the distinct segregation trends could be observed more intuitively (Figure 4).

**Table 3.** Differential metabolites between VF and non-VF groups

m/z	RT(min)	Metabolite	Metabolic pathway	VF vs. non-VF *
520.34	7.00933	LysoPC(18:2(9Z,12Z))	Phospholipid metabolism	–
522.356	7.61744	LysoPC(18:1(9Z))	Phospholipid metabolism	–
544.34	6.96251	LysoPC(20:4(5Z,8Z,11Z,14Z))	Phospholipid metabolism	–
546.355	7.27006	LysoPC(20:3(5Z,8Z,11Z))	Phospholipid metabolism	–
510.355	8.02127	LysoPC(17:0)	Phospholipid metabolism	–
457.232	6.80351	LPA(18:2(9Z,12Z)/0:0)	Phospholipid metabolism	–
506.36	8.92995	LysoPC(P-18:1(9Z))	Phospholipid metabolism	–
494.324	6.78781	LysoPC(16:1(9Z))	Phospholipid metabolism	Up
568.34	6.91188	LysoPC(22:6(4Z,7Z,10Z,13Z,16Z,19Z))	Phospholipid metabolism	Up
338.267	6.30823	Dehydrophytosphingosine	Phospholipid metabolism	Down
518.326	6.63885	LysoPC(18:3(9Z,12Z,15Z))	Phospholipid metabolism	Up
318.24	7.29255	9-cis-Retinoic acid	Retinol metabolism	Down
569.314	4.35167	Protoporphyrinogen IX	Porphyrin and chlorophyll metabolism	–
146.059	1.94401	1 H-Indole-3-carboxaldehyde	Porphyrin and chlorophyll metabolism	Up

*Compared with non-VF group, –: No significant difference. LysoPC lysophosphatidylcholines; LPA lysophosphatidic acid. RT Retention Time. The statistic significance was evaluated by calculating P values using the Student’s *t*-test.

### Functional analysis of the metabolic pathways

**Figure 5.**
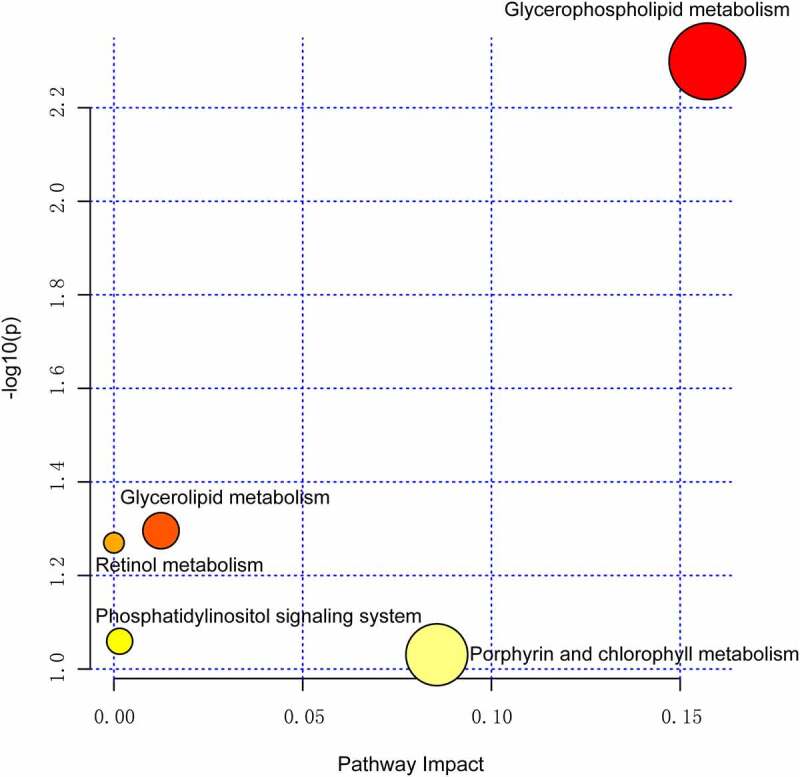
Pathway analysis summaries from MetaboAnalyst 5.0. All involved pathways are displayed as circles.

We further analyzed the metabolic pathway analysis, which showed that the metabolites identified for VF discrimination involve the metabolic pathways of glycerophospholipid, glycerolipid, retinol, phosphatidylinositol, porphyrin, and chlorophyll. Importantly, the metabolic pathway of glycerophospholipid was found to be the most dramatic pathway and possessed the strongest impact on the VF phenotype (*p* = 0.005, false discovery rate = 0.42, Figure 5).

### Biomarker identification and capability evaluation

**Figure 6.**
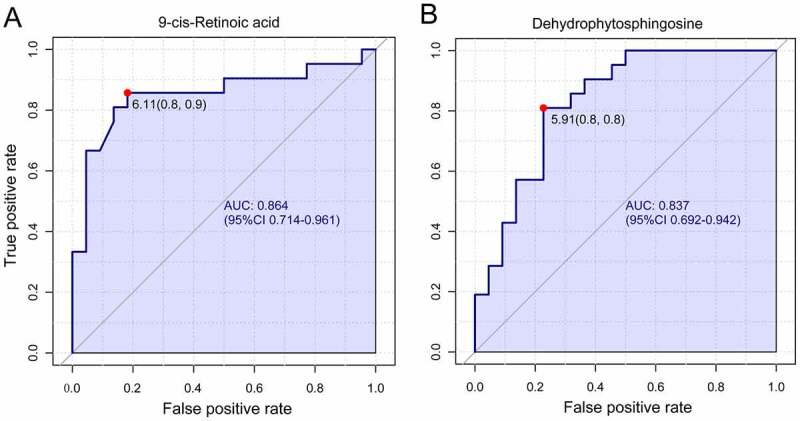
Univariate receiver operating characteristic curve for biomarker identification showed that 9-cis-retinoic acid and dehydrophytosphingosine were the two most important biomarkers, the area under the curve was 0.864 and 0.837 respectively.

The receiver operating characteristic curve (ROC) analysis was performed on the identified biomarkers, demonstrating that 9-cis-retinoic acid (9cRA) and dehydrophytosphingosine occupy the first two biomarkers, with values of area under the curve (AUC) of 0.864 and 0.837, respectively (Figure 6). The corresponding cutoff values (logarithmic conversion) and the associated sensitivity and specificity values were 6.11(0.8, 0.9) and 5.91(0.8,0.8).

**Figure 7.**
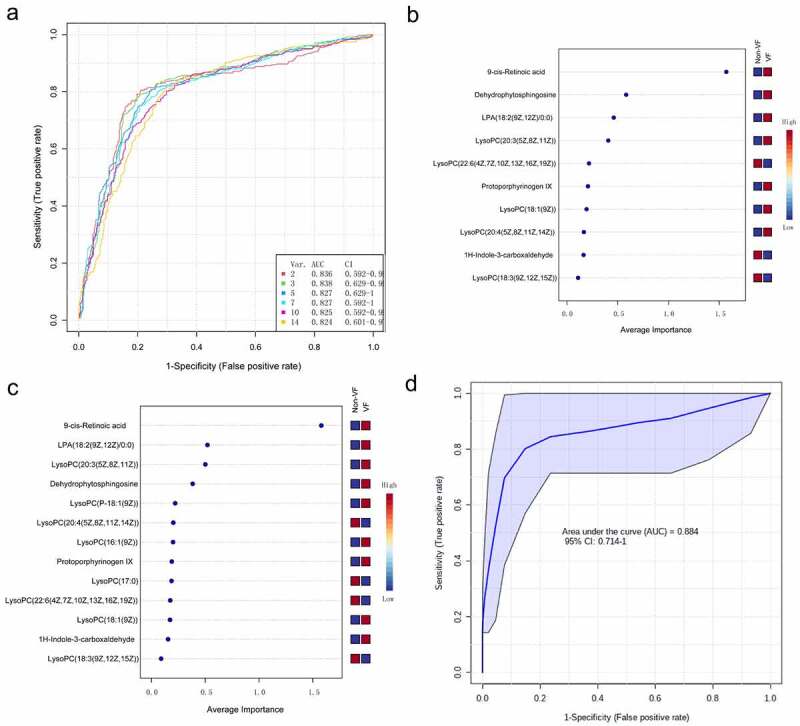
The process of feature screening, model construction, and performance assessment performed by Monte-Carlo cross-validation via MetaboAnalyst 5.0 (a) ROC curves of all models on the base of cross-validation performance. (B and C) In the model construction and performance assessment of biomarker prediction based on Monte Carlo cross-validation, significant biomarkers were ranked according to their frequencies of selection in Model 1 (b) and Model 2 (c). 9cRA was found to have the highest probability of appearing in these two models during cross-validation. (d) A ROC curve based model evaluation was conducted, in which the combination of 9cRA and dehydrophytosphingosine were selected.

Then, a multivariate ROC curve based on exploratory analysis was constructed for feature screening, model construction, and performance assessment. The SVM for classification and ‘SVM built-in’ for feature ranking was performed as a multivariate algorithm to identify biomarkers. The ROC curves based on cross-validation performance suggested that Model 1 (2 features) and Model 2 (3 features) had the two strongest predictive capability of early VF onset after STEMI, with close AUCs of 0.836 and 0.838, respectively (Figure 7a). Next, we built a biomarker-prediction model based on Monte Carlo cross-validation, and performance evaluation indicated that 9cRA has the greatest chance to enter the biomarker panels of Model 1 and Model 2 (Figure 7b & 7c), suggesting it served as the most important biomarker in our biomarker panel.

Finally, a ROC curve-based model evaluation was conducted. In this part of the test, any combination of features to create biomarker models could be manually selected based on the researchers’ own judgment or prior knowledge, and the capacity to predict the endpoint was again evaluated using the linear SVM algorithms. Here, we manually selected the combination of 9cRA and dehydrophytosphingosine, the top two features based on their overall ranks of AUC in univariate and multivariate ROC analysis, to create a biomarker model. As a result, the model was shown to have a good predictive value for early VF after STEMI, with an AUC of 0.884(0.714–1).

## DISCUSSION

The prompt revascularization via the widespread use of reperfusion strategies, such as primary percutaneous coronary intervention and drug therapy, has greatly improved the prognosis of patients with STEMI [17]. However, STEMI complicated by ventricular arrhythmias, mainly VF, remains a great concern as it can significantly increase the risk of mortality, as it may precede sudden cardiac death. Even with interventional therapy, in-hospital mortality rates of STEMI complicated with VF remain high [18,19]. It is therefore of paramount importance to ascertain methods for early identification of those at risk of VF and manage them in the first instance, expectantly improving the long-term prognosis.

Scar tissues that remain after a myocardial infarction are usually more likely to be arrhythmogenic than normal tissue due to regional changes in transmembrane action potential and refractory periods. This emphasizes that the presence of scar tissue from previous ischemic injury could further complicate a current STEMI and result in VF [20]. However, we know very little about how to characterize patients with no history of myocardial infarction and who were at high risk for developing VF at the early stage after the onset of STEMI. Acute myocardial infarction and the ensuing ischemia cause the death of cardiomyocytes due to imbalances between oxygen supply and demand, resulting in changes in the biochemistry of the myocardium. These events rapidly change the electrophysiological properties of the ventricular myocardium, which in turn leads to electrical disturbances in the conduction and repolarization of the ventricles, causing ventricular arrhythmias [21]. Therefore, the factors that determine the area of myocardial infarction (such as LMCAD as a culprit vessel) and serum potassium level in the early stage of morbidity may contribute to malignant arrhythmia after STEMI. However, other mechanisms that underpin these sequelae are still debatable. For example, with familial sudden death being a known risk factor for VF [22], there may be a genetic predisposition to higher risk. With this in mind, several genes have been researched in an attempt to establish whether they contribute to severe VF pathogenesis post-myocardial infarction. Genes associated with ion channels such as the *SCN5A* [23,24] and the *KCNJ11* genes [25] among other ion channel gene promoters [26] were thought to be associated with pathogenesis of VF and sudden cardiac death. However, some of these genes were found to have little to no association and remain a topic of discussion. Nonetheless, genetics alone do not predict phenotypic presentation due to the impact of environmental influences and epigenetic alterations. In the analysis of the clinical data of the patients enrolled, we found that the patients with LMCAD had a higher (but not significant) incidence of early VF (13.6% vs 9.3%), and the proportion of culprit vessels, left main coronary artery and left anterior descending branch, in the early VF group was significantly greater than that in the non-VF group (*P* = 0.021). When the PSM was performed to adjust for clinical variables, including the distribution of culprit vessel, however, it was found unexpectedly that the proportion of early episodes of VF in the LMCAD group was significantly lower than that in the non-LMCAD group (4/22 vs. 17/22, *P* < 0.001). This indicated that LMCAD significantly contributes to the occurrence of VF early after STEMI, but it is not the only decisive factor. Other independent etiologies may also exist.

Metabolomics is the study of metabolites in biological samples, which can provide a functional view of organisms under the combined effects of genetics and environmental influences. To the best of our knowledge, this is the first published study on metabolic profile analysis in patients with early VF after STEMI. Thus, this technique of study may be paramount to finding useful predictors of VF post-myocardial infarction and may become the basis of great clinical benefit, reducing mortality and for adapting procedural processes such as transport between centers and prioritization of patients.

The mainstream metabolomic analysis platforms include gas chromatography-mass spectrometry (GC-MS) and liquid chromatography-mass spectrometry (LC-MS). The two platforms have their own advantages, which are mainly determined by the physical and chemical properties of the target compounds. GC-MS is suitable for volatile compounds with low molecular weight. The ion source is electron ionization. A large amount of ion fragment information can be obtained, and there is a mature metabolite library for compound retrieval. For nonvolatile components, derivatization is required; LC-MS ion source is an atmospheric pressure chemical ionization or electrospray ionization source. It is suitable for the detection of compounds with medium or strong polarity with a wide molecular weight range, high sensitivity and requires simple pre-treatment. Based on these considerations, LC-MS platform was applied in this study.

As mentioned above, considering the inter-group heterogeneity of these clinical indicators potentially related to the occurrence of VF will interfere with the determination of differential metabolites between groups, we used propensity score matching for patient enrollment to reduce the adverse impact. The determination of adjusted variants mainly comes from the consensus of cardiovascular experts in our group, including grading of cardiac function, serum potassium and magnesium levels, the location of criminal blood vessels, etc. Although there are other potential clinical factors that may affect the morbidity of VF, the inclusion of too many correction variants can negatively affect the number of matched subjects that can be obtained.

The time of blood collection is a very important consideration. Sampling times vary widely in the previous publications. In these studies, blood collection times included overnight fasting, immediately after admission (non-fasting), and at different points after admission. The ideal sampling time should be when the metabolic state is stable. However, patients with STEMI are always in a state of metabolic deviation. Therefore, there is no recognized and absolutely ideal blood collection time point for them. The purpose of our study is to identify the differential metabolites of ventricular fibrillation in the early stage after the onset of STEMI. Therefore, we believe that the time point of blood collection should be as early as possible, and in a clinical sense, the scenario of ventricular fibrillation after coronary revascularization is not the core issue of this study. In the latter cases, patients are often in a close monitoring state of vital signs, and the probability of sudden death is far less than that in the pre-hospital/emergency settings before intervention.

Using UPLC/MS, a high-throughput technology used in metabolomics to analyze differentially expressed metabolites, we found fourteen significantly altered metabolites in the VF group compared to the non-VF group, specifically 9cRA and dehydrophytosphingosine. Through further pathway analysis, the identified target metabolites were related to pathways concerning the metabolism of glycerophospholipid, glycerolipid, retinol, phosphatidylinositol and porphyrin and chlorophyll. We suggest that at least one, if not several, of these metabolites could potentially help reveal the mechanism behind VF and may prove to be an important predictive biomarker. This may lead to improved clinical outcomes.

As expected, the glycerophospholipid metabolism pathway was the most important altered pathway and some of the differential metabolites in the pathway, e.g., lysophosphatidylcholine (LysoPCs), accounted for the largest weight for the VF phenotype. Compared to the non-VF group, the levels of various types of LysoPCs were shown to be significantly increased in the post-MI VF group. LysoPCs, an important component of cell membrane, are released into the blood following cell necrosis and cell membrane disintegration after myocardial infarction, resulting in a significant increase in its serum level. This explains why previous metabolomics studies by us and other groups in patients with different characteristics of myocardial infarction – such as ischemia reperfusion injury [27], young morbidity [12] and LMCAD subtype [14] – generally demonstrate that the glycerophospholipid metabolism pathway has become the most significantly changed pathway. This is determined by the pathological characteristics of myocardial infarction itself. However, this pathway has not distinguished the features we are interested in.

In the multivariate ROC curve based on exploratory analysis, a model with 3 metabolites (9cRA, dehydrophytosphingosine and lysophosphatidic acid (18:2(9Z, 12Z)/0:0)) yielded the highest AUC of 0.838. This was closely followed by a 2-metabolite model (9cRA and dehydrophytosphingosine) with an AUC of 0.836. In the prediction models of biomarkers, the AUC is an index of diagnostic capability. The higher the AUC, the greater the relative diagnostic capability toward the VF phenotype. Of the metabolites we identified, 9cRA was found to be the most valuable of all the biomarkers, and it was most likely to be present in the predictive panel of biomarkers. The metabolites in the model were found to be involved in a diversity of pathways involving vitamin A metabolism (9cRA), glycerophospholipid metabolism (dehydrophytosphingosine) and glycerolipid metabolism (lysophosphatidic acid (18:2(9Z, 12Z)/0:0)). We believe that disruption of at least one of these pathways leads to the onset of ventricular fibrillation. Furthermore, we found that the level of 9cRA in the VF group was lower than that of the non-VF group (Table 3). 9cRA, which is a derivative of retinol, was shown to have the most important discriminative properties for the phenotype and was found to have highest probability of being selected by the SVM feature selection algorithm. From this, we conclude that retinol metabolism dyshomeostasis is significantly associated with VF post-myocardial infarction.

The retinol metabolic pathway is composed of retinoic acid, (intracellular) retinol/retinoic acid binding protein, retinoic acid receptors (RARs), retinoid X receptors (RXRs), cis-retinoic acid responsive element and target genes. Retinoic acid is an indispensable substance for maintaining growth and development, which is mainly involved in regulating cell proliferation and differentiation. It has a wide range of biological activities. In the past decade, there has been a better perception of the signaling pathway, particularly the interrelation between its systemic homeostasis and gene regulatory network. LncRNA Tsix was found to specifically bind *mir-34a-5p*, which relieves the inhibition of retinol binding protein 2 expression by the latter, plays a role in anti-cardiomyocyte apoptosis and improves cardiac function injury after myocardial infarction [28]. All-trans-retinoic acid was shown to suppress rat embryo hindlimb bud mesenchymal chondrogenesis. 9cRA is an isomer of all-trans retinoic acid. As a differential metabolite, the expression level of 9cRA significantly decreased in the process of breast cancer progression and metastasis [29]. Using UPLC/MS, we have identified that retinol metabolism is the most valuable metabolic pathway in differentiating the morbidity of LMCAD [14]. We also found that 9cRA possessed the most discriminative power as a predictor among a ten-metabolite plasma biomarker panel. RARs and RXRs are two classes of nuclear receptors, which are found to be related to the 9cRA expressing its target genes. The latter exists as a liable heterodimer for several receptors that perform different functions including liver X, pregnane X, peroxisome proliferator activated, vitamin D, farnesoid X and thyroid hormone [30]. This contributes to the diversity of 9cRA and demonstrates the way its dyshomeostasis could influence various pathological processes in the case of disruption of associated RXRs. Although there is a discussion around their role, RXRs have been shown to influence development, metabolic diseases, cancer, and atherosclerosis [31,32]. Moreover, RXR motifs are also present in many potassium voltage-gated channels [30]; given that a well-established electrophysiological change in an infarct border zone includes a reduction in repolarizing K^+^ currents, it could be a case that dyshomeostasis of 9cRA disrupts the functioning of RXR motifs affecting currents across the cell membrane of myocardium rendering it susceptible to aberrant conduction pathways.

Dehydrophytosphingosine is part of the sphingolipid class of lipids. Sphingolipids are one of the components of biomembranes involved in various physiological functions such as glucose metabolism, neural functions, cell adhesion, immunity, and skin barrier [33–35]. Dehydrophytosphingosine is a precursor of ceramide, which is the core member of the sphingolipid metabolic pathway and is involved in the cellular response to ischemic stress or ischemia reperfusion injury and in the activation of exogenous and endogenous apoptotic pathways. Dehydrophytosphingosine, as a lipid barrier and natural inflammation regulator, plays an anti-inflammatory and antibacterial role and effectively inhibits a variety of inflammatory cytokines, including interleukins 1 alpha, protein kinase C, etc., mainly by inhibiting the synthesis of prostaglandins [36]. A better understanding of the pathways involved in the lack of production of dehydrophytosphingosine in the VF group could help in elucidating treatment targets for the prevention of the development of VF. Recent studies display that sphingolipids are biomarkers of recurrence and mortality following MI [36,37]. It has been reported that there is a change of the sphingolipid profile following an MI and the sphingolipid metabolism is correlated with cardiac pathophysiology, particularly concerning levels of ceramide [38]. Furthermore, perturbed sphingolipid metabolism resulted in arrhythmias and other cardiac dysfunction in animal mice models [38,39].

Considering the diagnostic capabilities displayed, the novel biomarker panel offers great opportunities in the prediction of early VF post-STEMI and potential avenues of treatment strategies. These metabolic changes and the related pathways provide novel insights into the morbidity of VF. We propose that disruption to the pathways could possibly lead to abnormal activity either in the ion channels of the heart or even irregularities in cardiac remodeling during the reparative phase post-AMI. Nevertheless, elucidating the association between the metabolites, metabolic pathways, and pathological mechanisms could aid in understanding the pathophysiology behind VF post-MI.

This study has some limitations. Although we used a PSM approach to balance baseline parameters between pairs that could otherwise affect the accuracy of test results, we were only able to perform our analysis on a relatively small number of samples. This creates a risk of overfitting, and thus the predictive capabilities of the two metabolites derived need to be further tested in larger, more varied cohort samples. Due to lack of external validation, the adaptability of the model is yet to be determined. Moreover, we note that the biomolecules contained in extracellular vesicles in bio-fluids are being used as emerging biomarkers, which have great potential significance for clinical diagnosis and disease prognosis, such as tumor and microbial infection [40–42]. This is due to the recent development of extracellular vesicle separation and characterization technology [43]. The future clinical transformation research in the cardiovascular field will be one of the facets we will pay attention to.

## Conclusion

By means of a UPLC/MS platform, retinol metabolism was determined to be the most significant discriminative pathway for VF in the early stage after STEMI. A 2-metabolite model (9cRA and dehydrophytosphingosine) may function as a potential predictive model for evaluating the reproducibility of the VF phenotype, in which 9cRA has a robust discrimination ability. Overall, 9cRA and dehydrophytosphingosine showed the strongest levels of discriminative power in the prediction of early VF post-STEMI, but further research is required to assess reproducibility.
